# O023: Combining electronic contacts data and virological data for studying the transmission of infections at hospital

**DOI:** 10.1186/2047-2994-2-S1-O23

**Published:** 2013-06-20

**Authors:** C Payet, A Barrat, C Cattuto, C Régis, N Khanafer, J-F Pinton, B-A Kim, B Comte, B Lina, P Vanhems, N Voirin

**Affiliations:** 1 Université Lyon 1, CNRS UMR 5558, Lyon, France; 2 CNRS, CPT, UMR 7332, Marseille, France; 3 Data Science Lab, ISI Foundation, Turin, Italy; 4 Hospices Civils de Lyon, France; 5 CNRS UMR 5672, France; 6 Hôpital Edouard Herriot, Service de gériatrie, France; 7 Université Lyon1, CNRS FRE 3011, Lyon, France

## Introduction

Transmission of hospital acquired infections (HAI) depends mainly on contacts between patients, between health care workers (HCWs) and between patients and HCWs.

## Objectives

The objective of this study was to combine contacts data and virological data for studying influenza transmission during an outbreak occurring in a hospital unit.

## Methods

Face-to-face proximity between persons was collected during 10 consecutive days using electronic RFID badges. Virological data on influenza infection status were also collected. Each patient and each HCW had 2 nasal swabs, one at admission and one at discharge for patients, and 2 swabs at 7 days interval for HCWs, from which laboratory confirmation of influenza infection was performed.

## Results

A total of 18,766 contacts were recorded among 37 patients and 47 HCWs. Nurses, medical doctor( MD) and patients were involved in 82%, 26% and 24 % of all the contacts respectively. In parallel, during the 10 days, an outbreak occurred involving 15 laboratory-confirmed influenza cases diagnosed among 10 patients (attack rate 27%) and 5 HCWs (attack rate 10%). We identified 5 (14%) patients and 10 (20%) HCWs who cumulated nearly 50% of all the contacts involving patients and HCWs. Among these persons with a high number of contacts, 3 (60%) patients and 1 (10%) HCW had confirmed influenza. Among those with a lower number of contacts, 7 (22%) patients and 4 (11%) HCWs had confirmed influenza. Further statistical analyses are ongoing to assess the relationship between the number and duration of contacts and the risk of influenza transmission.

## Conclusion

Collecting contacts data in the hospital setting and combining this information with virological data could be an interesting approach to study the transmission of HAIs. We identified patients and HCWs with a high number of contacts, who could be considered as potential super-spreaders of infections. This is key information that may help to implement prevention and control measures.

## Disclosure of interest

None declared.

